# Designing a Score-Based Method for the Evaluation of the Nutritional Quality of the Gluten-Free Bakery Products and their Gluten-Containing Counterparts

**DOI:** 10.1007/s11130-018-0662-5

**Published:** 2018-04-25

**Authors:** Federico Morreale, Donato Angelino, Nicoletta Pellegrini

**Affiliations:** 1 Human Nutrition Unit, Department of Food and Drug, University of Parma, Parco Area delle Scienze 47/A, 43124 Parma, Italy; 2 Food Quality and Design Group, Wageningen University, Wageningen, the Netherlands

**Keywords:** Nutritional quality, Gluten-free bakery products, Score-based method, Celiac disease

## Abstract

Gluten-free (GF) products are consumed both by individuals with celiac disease and by an increasing number of people with no specific medical needs. Although the technological quality of GF products has been recently improved, their nutritional quality is still scarcely addressed. Moreover, the few published studies report conflicting results, mostly because the information from product nutrition facts is the only considered factor. The aim of the present study was to develop a score-based method for the nutritional evaluation of 134 packaged Italian GF bakery products and to compare it with that of 162 matched gluten-containing (GC) food items. The score included the information from the nutrition facts and the presence/absence of some nutritionally relevant components in the ingredients list. Results indicated an overall low nutritional quality of the considered GF bakery products. Additionally, with the sole exception of GF bread substitutes, there was no difference in nutritional quality between GF and equivalent GC bakery products. Future research and development of GF bakery products may take advantage of this scoring method, as it may represent an easy approach to evaluate their nutritional quality. The present findings do not justify the consumption of packaged GF bakery products by people without any specific medical needs.

**Electronic supplementary material:**

The online version of this article (10.1007/s11130-018-0662-5) contains supplementary material, which is available to authorized users.

## Introduction

The total exclusion from the diet of foods containing gluten is the only possible treatment for celiac disease (CD), an autoimmune disorder sustained by an inappropriate response to gluten ingestion in genetic predisposed individuals [1]. A gluten-free (GF) diet includes naturally GF foods, such as vegetables, fruits and meat, and GF products developed to substitute the traditional cereal-based foods. It has been estimated that at least 5% of the world population needs to follow a GF diet for medical purposes [2], although a specific medical need is not an essential reason to follow it. Furthermore, the GF diet has recently become a cultural phenomenon involving the search for foods free of one or more ingredients that are supposed to be unnatural or unhealthy [3]. Consequently, the GF market has recently seen a remarkable growth, with sales of GF foods increased about by 136% between the 2013 and 2015 in the US, reaching a total value of around $11 billion [4]. In Europe, the latest economic reports foresee a regular growth rate of about 10% until 2019 [2].

Owing to the growing interest in GF products, their formulation and production processes have been recently put under the spotlight, with a particular attention towards GF bakery products. However, all these efforts in GF product development and/or improvement have been mainly focused on the technological and sensory aspects, leaving the nutritional quality very poorly addressed [5]. To overcome the technological constraints associated to the absence of gluten, and therefore improve the texture and the sensory characteristics of GF products, various food additives and co-texturizers are applied [6]. These ingredients affect the nutritional quality of such products.

Despite a growing popular perception that GF products are healthier than the gluten-containing (GC) counterparts, their real nutritional quality is still far to be conclusively defined. Actually, a limited number of conflicting studies have assessed the nutritional quality of GF products and compared it to that of their GC counterparts. Some authors [7, 8] have reported a higher content of total and saturated fat in GF products, whereas others [9, 10] have found no differences between the two types of product in terms of such nutrients. In addition, inconsistent results about the content of dietary fiber have been reported [8, 9]. Such discrepancies in nutritional quality definition of GF products may also be attributable to the high variation of GF formulations and/or to a low ability of the methods used to measure the nutritional quality.

To try to partially address this issue, and referring to the Italian market of GF products, we have developed a score-based method in order to assess the quality of packaged GF products and to compare with that of similar GC counterparts. The focus of this work is on the bakery products as they represent staple foods largely consumed and important sources of nutrients for the general population.

## Materials and Methods

### Selection of the Products

According to the latest trends in sales of the Italian food market (2015), kindly provided by Dr. Schär GmbH/Srl, packaged products from the most representative Italian brands (almost 60% of the market sales) producing GC and/or GF bakery foods were selected for the present study. GF bakery products and their GC counterparts were grouped into four food categories: bread, bread substitutes, cookies and breakfast pastries. The list of the type of products analyzed in each food categories is reported in Online Resources (Table 1S). Information about the nutritional composition and ingredients was directly collected on both the food manufacturer’s website and the product pack.

### Design and Application of a Score-Based Method

We developed the score-based method by considering two groups of parameters: i) amount of specific macronutrients and ii) nutritional quality of some ingredients in the food formulation.

The first group of parameters was quantitative, and included total and saturated fat, sodium, fiber and sugar. Their reference amount was selected according to the annex *“Nutrition claims and conditions applying to them”* of the EU regulation No 1924/2006 [11]. The quantification was based on the nutrition facts information available on the food pack label, and the relative amount of such parameters was scored with points from 0 to 2, as described in Table 1. The overall sum may reach up to 7 points.

**Table 1:** Considered information from nutritional facts of products and points assignment for the quantitative part of the score calculation^a^

Parameters	Zero points	One point	Two points
Total fat (g/100 g)	>3	<3	
Saturated fat (g/100 g)	>1.5	<1.5	
Sodium (g/100 g)	>0.4	<0.4	<0.12
Fiber (g/100 g)	<3	>3	
Sugar (g/100 g)	>5	<5	<0.05

^a^According to the limits stated in the Regulation (EC) No 1924/2006 – *Annex “Nutrition claims and conditions applying to them”*

The second component of the score was qualitative and designed to emphasize the presence or absence of specific ingredients in determining the overall nutritional quality of the considered products. The qualitative parameters were selected according to the recent proposed strategies to improve the nutritional quality of the GF bakery products [12–14]. In particular, as described in Table 2, the presence/absence (yes/no) of the following ingredients was evaluated: i) starch as first or principal ingredient; ii) wholegrain flours; iii) sourdough (only as a leavening agent); iv) flour from legumes; v) other flours, from minor cereals and/or pseudocereals (*i.e.*, buckwheat, quinoa, amaranth and sorghum, used as alternative to wheat or traditional GF cereals); vi) fructose; vii) emulsifiers (mono and diglycerides of fatty acids). The score for each product was obtained by summing the points assigned to the amount of specific nutrients (quantitative parameters) and the points resulted from the qualitative parameters. As the number of qualitative parameters used to describe each food category was different, the maximum score was different among food categories. In particular, for bread and bread substitutes the score ranged from 0 to 13 points, for breakfast pastries from 0 to 12 points, and for cookies from 0 to 11 points.

**Table 2:** Considered nutritionally relevant ingredients and points assignment for the qualitative part of the score calculation^a^

Parameters	Zero points	One point
Starch as first ingredient	Yes	No
Wholegrain flours	No	Yes
Sourdough^1^	No	Yes
Flour from legumes	No	Yes
Other flours^2^	No	Yes
Fructose^3^	Yes	No
Emulsifiers^4^	Yes	No

^a^Points were assigned according to the presence/absence (yes/no) of the ingredients

^1^only bread; ^2^ gluten-free ingredients different from rice and corn, such as buckwheat, quinoa, sorghum, etc. and gluten-containing cereals different from wheat, such as rye and barley; ^3^in the form of corn syrup in cookies and breakfast pastries; ^4^mono- and diglycerides of fatty acids

### Statistical Analyses

Shapiro-Wilk test was used to evaluate the normality of distributions. The score obtained for the GF bakery products was compared to that obtained for the GC counterparts by means of the Mann-Whitney test. To determine whether the score method misclassified the considered products, a further evaluation by means of the Mann-Whitney test based only on the quantitative parameters was performed. All data analyses were performed by using IBM SPSS® Statistics software 22.0 (IBM Corp., Chicago, IL). Significance was accepted at *p* < 0.05.

## Results and Discussion

The evaluation of the nutritional quality of GF products has been mainly based on the information retrievable on nutrition facts [8, 10, 15]. Nevertheless, the nutritional quality of a bakery product cannot be only ascribed to its macro- and micro-nutrient content. For instance, the inclusion of flours rich in dietary fiber in the formulation of bread products, *e.g.*, those obtained from amaranth, quinoa or buckwheat, is a common practice [16]. However, these flours may influence more than the only content of dietary fiber. Indeed, they allow to partially replace ingredients such as starch from potato or cassava and refined flours present in the formulation, thus improving the content of several nutrients scarcely contained in GF bakery products, e.g., proteins, various vitamins and minerals [16].

**Fig. 1:**
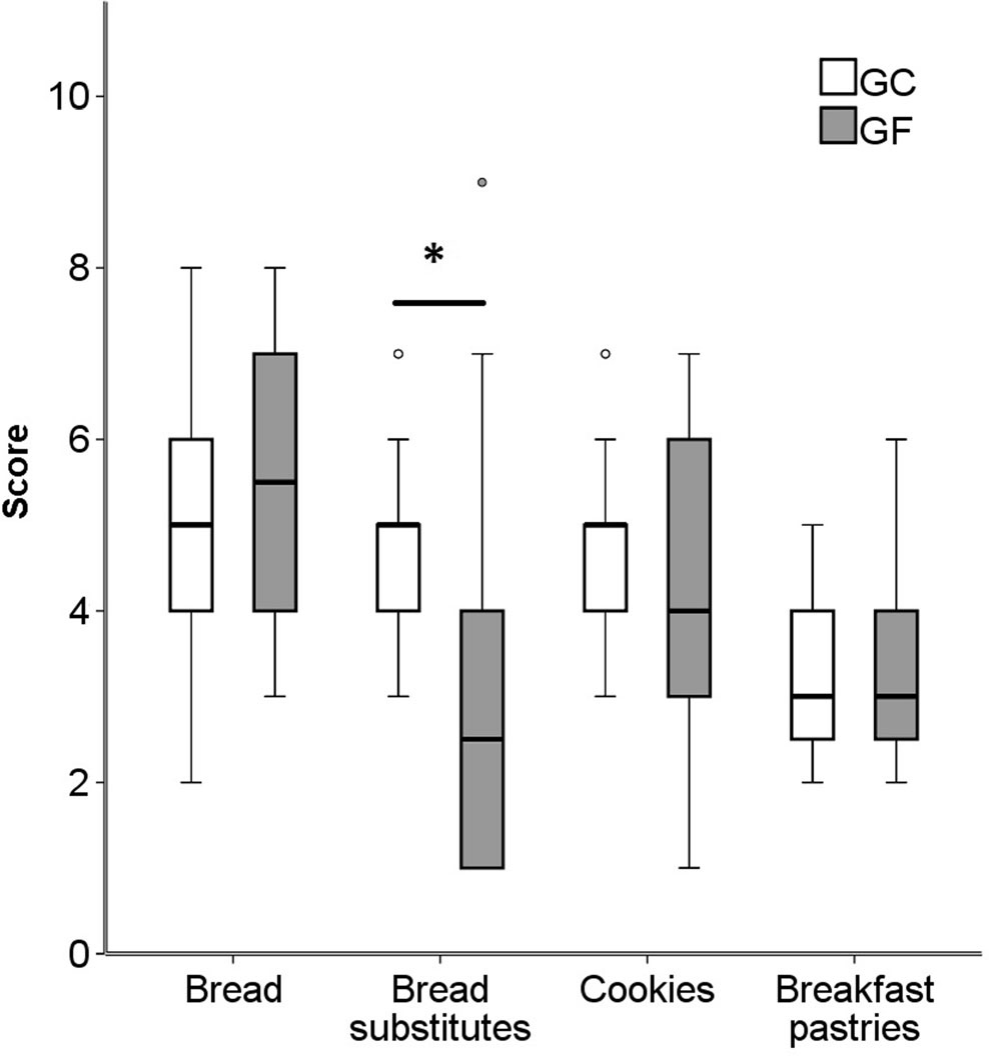
Box-plot graphs showing the score of the GF products compared to that of the GC counterparts. For bread and bread substitutes, the score ranged from 0 to 13 points, for breakfast pastries from 0 to 12 points and for cookies from 0 to 11 points. (*) indicates a significant difference, Mann-Whitney, *p* = 0.001

In this study, a total of 134 Italian packaged GF and 162 GC bakery products, grouped into four food categories, were evaluated using a nutritional quality score-method. This score considered not only the information from nutrition facts, but also the contribution of some nutritionally relevant components in the ingredients list. Applying this score, an averagely low nutritional quality of the considered GF bakery products emerged, as shown in Fig. 1. Interestingly, GF bread, cookies and breakfast pastries scored relatively close to their GC counterparts. The only clear exception was GF bread substitutes, which showed a significantly lower nutritional quality when compared to their GC counterparts (*p* = 0.001).

The findings of the present study are in agreement with those reported by Wu et al. [9], who compared the nutritional quality of several Australian packaged GF products, across ten food categories, to their matched GC counterparts. The nutritional quality of GF products was based on a descriptive score, namely the “Health Star Rating” (HSR) system. The HSR system is a combination of some baseline points, taking into account the amount of saturated fat, total sugar and sodium, and of several points attributed by the presence of specific food components, including fruit, nuts, vegetables, legumes, and the content of protein and dietary fiber. Authors evidenced that the GF bakery products in several food categories, such as bread, cakes, and cookies, were not significantly different in their nutritional quality when compared to GC similar items.

Our results are instead in disagreement with those of Miranda et al. [8] and of Kulai and Rashid [15], who considered only the nutrient content. The first study evidenced a significantly better nutritional profile of GC in comparison to GF products in terms of the content of energy, saturated, and total fats. In the study of Kulai and Rashid [15], the GC breads showed better nutritional value than GF substitutes, as the latter were significantly higher in total fat and lower in protein.

Despite the low nutritional quality portrayed by the score, some attempts at improving the nutritional quality of GF bakery products emerged (Table 3) from our observations.

**Table 3:** Percentages of products, divided into food categories, matching the conditions^1^ used to calculate the score

Food category		Low fat	Low saturated fat	Low sodium	Source of fiber	Sugar free	Low sugar	Starch as first ingredient	Wholegrain flours	Sourdough	Flour from legumes	Other flours	Fructose	Emulsifiers
		%	%	%	%	%	%	%	%	%	%	%	%	%
Bread	GC^2^ (*n* = 34)	9	62	0	76	0	50	0	38	21	12	47	n.u	15
	GF (*n* = 24)	4	67	0	88	0	88	58	4	54	67	79	n.u	63
Bread substitutes	GC (*n* = 49)	2	16	2	88	0	96	0	16	n.u	0	47	n.u	8
	GF (*n* = 26)	8	12	0	46	12	62	77	0	n.u	19	27	n.u	59
Cookies	GC (*n* = 43)	0	5	28	56	0	9	2	0	n.u	7	n.u	28	2
	GF (*n* = 53)	0	0	30	42	0	0	42	0	n.u	49	n.u	6	28
Breakfast pastries	GC (*n* = 36)	3	8	8	17	0	6	0	0	n.u	0	n.u	53	89
	GF (*n* = 32)	0	0	3	25	0	6	38	0	n.u.	28	n.u.	19	63

^1^Regulation (EC) No 1924/2006 (quantitative parameters); presence/absence of nutritionally relevant ingredients (qualitative parameters). n.u means that the ingredient is not used. ^2^ GC, gluten-containing; GF, gluten-free

Starch is one of the most relevant ingredients deeply affecting nutritional quality of GF bakery products. Due to its bland taste, starch presence as first or main ingredient entails salt and lipid addition to GF bakery products in order to enhance their low palatability [12]. Table 3 shows that in 42% of considered GF bread formulations starch was not the first or principal ingredient.

The main strategy for reducing starch content in bakery products is its partial substitution with flour obtained from nutritionally valued minor cereals and pseudocereals, especially in GF bread making [17, 18]. Among these alternative ingredients, quinoa, buckwheat, and sorghum have attracted attention because of their nutritional composition, providing relevant amounts of dietary fiber, B-vitamins and iron [16, 19]. Interestingly, our results confirmed that this enrichment trend involves several GF breads, as 79% of the evaluated products contained flours from minor cereals and/or pseudocereals, and the 88% could be labelled as “source of fiber” according to the Regulation (EC) No 1924/2006 (Table 3). This data seems to disagree with the general belief that GF bakery products scarcely contribute to the daily intake of dietary fiber [20].

In the last few years, the sourdough fermentation has been introduced in the production of industrial GF bread. In GF products, the sourdough is composed of a wide range of GF flours (rice, corn, buckwheat, etc.) and water, and is fermented by yeasts and lactic acid bacteria (LAB) [21]. LAB produce long-chain polysaccharides that may act as a co-adjuvant of the common hydrocolloids used in GF bread making [21]. In view of this, the sourdough employment seems to fulfil more a technological purpose rather than a nutritional enhancement. However, these long-chain polysaccharides contribute to the daily intake of dietary fiber, and may behave as prebiotics [22]. In fact, some studies have shown that these polysaccharides may be fermented by the intestinal microbiota and in turn modulate the immune response [23]. Considering our results, sourdough was present in 54% of GF breads formulations compared to 21% of the GC breads. In contrast with some improvements emerged in GF bread production, the nutritional profile of GF bread substitutes resulted inadequate. Starch was not the first ingredient in only the 23% of GF bread substitutes and no wholegrain flour was included in their formulations (Table 3). Flours obtained from other cereals were included in 27% of GF bread substitutes, against the 47% of the GC similar products. As a consequence, only the 46% of GF bread substitutes could be labelled as “source of fiber”, with respect to the 88% of their GC counterparts (Table 3). To date, bread substitutes represent a substantial part of the sales of GF bakery products [24] and they are often consumed as a snack or an alternative to bread by individuals with CD [25]. For this reason, great care should be taken to improve their nutritional composition.

Cookies and breakfast pastries, in general, are driven, in their formulation, by different marketing needs. Their content of sugar and total fat – but also the quality of these fats – is functional to ensure their specific texture, their palatability and, as a consequence, consumer acceptability [26]. Therefore, we did not expect GF cookies and breakfast pastries to be low in sugar, total and saturated fat. However, considering the positive results reported by some studies aiming to improve the nutritional value of these GF products [27, 28], we were expecting to identify more products containing whole grain flours and/or flours from minor cereals and pseudocereals at least.

Among the limitations of this scoring method there is its partially qualitative nature. Some parameters may have negatively affected the comparison between GF and GC products, since the score was mainly set-up to evaluate the nutritional quality of the GF bakery products. For instance, flours from legumes are often incorporated in GF bakery products to improve qualitative characteristics, such as viscoelastic functionality of dough, sensory acceptance and shelf-life [13], but they are hardly present in the formulation of GC bakery products. However, although the developed score method was designed for the nutritional evaluation of GF bakery products, the results did not change when the evaluation was based on the sole quantitative parameters (Table 2S). Also in this case, the considered Italian GF bakery products had a low nutritional profile similar to the GC counterparts. The only exception was for the GF bread substitutes, which obtained significantly less points than those of their GC counterparts (*p* = 0.005).

Another limitation of the present study concerns the exclusion of micronutrient content in the developed score. This is mainly attributable to the fact that, according to the European Regulation [29], information about micronutrient content is not mandatory in the nutrition fact. The only available data on micronutrient content in GF products comes from two studies that analyzed or estimated the concentration of minerals and vitamins in GF food products in the Polish and the Austrian market [10, 19].

The low nutritional quality of all the considered products (*i.e.*, GF and GC) may be partly explained by the fact that they were packaged food items. In this sense, it is worth to remind that some ingredients used in packaged bakery goods, such as emulsifiers and salt, cannot be completely avoided or reduced due to their role in both the GC and GF baking process [4]. For example, it is quite a challenge to produce sliced bread by lowering salt content below the value established as “low in sodium” by EU Regulation (EC) No 1924/2006 (*i.e.*, 0.12 g/100 g of sodium or the equivalent 0.3 g/100 g of salt), without affecting some important quality parameters, such as texture and shelf life [30].

## Conclusions

Based on the results of this study, the nutritional quality of the analyzed Italian GF bakery products resulted low and comparable to that of GC counterparts. Therefore, the present findings do not justify the consumption of packaged GF bakery products instead of the traditional GC ones by people without any specific medical need. Rather, this work suggests that the formulation of these products should be revised in order to improve their nutritional profile and to aim to the highest score possible. This means formulating an ideal GF bread by firstly avoiding starch as first or main ingredient. Moreover, the addition of wholegrain GF cereals and legumes to common GF flours will guarantee a nutritional improvement in terms of micronutrients. Sourdough should be preferred as a leavening technique. The developed scoring method could direct also food manufacturers in reformulating their products, as it may represent an easy approach to evaluate the nutritional quality of the GF bakery products. The further integration of the developed score with information about important micronutrients for individuals with CD, *e.g.*, calcium, iron, magnesium, zinc, would be useful to allow a more comprehensive nutritional evaluation.

## Electronic supplementary material

ESM 1 (DOCX 18 kb)

## Abbreviations

CD: Celiac disease
GC: Gluten containing
GF: Gluten-free
LAB: Lactic acid bacteria
HSR: Health star rating
