# Supplementary material for: Designing a Score-Based Method for the Evaluation of the Nutritional Quality of the Gluten-Free Bakery Products and their Gluten-Containing Counterparts
Source: Plant Foods Hum Nutr. 2018 Apr 25;73(2):154–9. doi: 10.1007/s11130-018-0662-5 (PMC5956026; doi:10.1007/s11130-018-0662-5)
Supplement: Supplementary file 1 — (DOCX 18 kb) [file 11130_2018_662_MOESM1_ESM.docx]

**Article Title:** Designing a score-based method for the evaluation of the nutritional quality of the gluten-free bakery products and their gluten-containing counterparts.

**Journal:** Plant Foods for Human Nutrition.

**Authors:** Federico Morreale, Donato Angelino, Nicoletta Pellegrini.

**Corresponding author:** Nicoletta Pellegrini, [nicoletta.pellegrini@unipr.it](mailto:nicoletta.pellegrini@unipr.it). Human Nutrition Unit, Department of Food and Drug, University of Parma, Parco Area delle Scienze 47/A, 43124 Parma, Italy.

**Table S1** Number and type of products included in each food category and sub-category.

| Categories | Sub-categories | Gluten-containing products | Gluten-free products | Total |
| --- | --- | --- | --- | --- |
| Bread (sliced) | White | 12 | 9 | 21 |
|  | Multigrain and seeds | 14 | 14 | 28 |
|  | Wholegrain | 8 | 1 | 9 |
|  | Total | 34 | 24 | 58 |
| Bread substitutes | Crackers: plain, savory | 21 | 14 | 35 |
|  | Crackers wholegrain | 9 | 0 | 9 |
|  | Breadsticks: plain, savory | 19 | 12 | 31 |
|  | Total | 49 | 26 | 75 |
| Cookies | Biscuits plain | 10 | 18 | 28 |
|  | Chocolate biscuits | 6 | 13 | 19 |
|  | Biscuits filled with milk or chocolate cream | 8 | 10 | 18 |
|  | Biscuits wholegrain | 5 | 1 | 6 |
|  | Wafer filled with milk, chocolate, vanilla cream | 14 | 11 | 25 |
|  | Total | 43 | 53 | 96 |
| Breakfast pastries | Plum cake: plain, yogurt, chocolate | 9 | 11 | 20 |
|  | Croissant filled with jam, cream, or chocolate | 17 | 4 | 21 |
|  | Sponge cake filled with jam, milk, chocolate cream | 7 | 14 | 21 |
|  | Muffin | 3 | 2 | 5 |
|  | Total | 36 | 31 | 67 |
|  | Total | 162 | 134 | 296 |

**Table S2** Comparison between GF products and GC counterparts based on the quantitative parameters.

|  |  | Score Percentiles | | |  |
| --- | --- | --- | --- | --- | --- |
| Categories |  | 25th | Median | 75th | *p*-value^1^ |
| Bread | GC | 1.00 | 2.00 | 3.00 | 0.06 |
|  | GF | 2.00 | 3.00 | 3.00 |  |
| Bread substitutes | GC | 2.00 | 2.00 | 2.00 | <0.01 |
|  | GF | 1.00 | 1.50 | 2.00 |  |
| Cookies | GC | 1.00 | 2.00 | 2.00 | 0.20 |
|  | GF | 1.00 | 2.00 | 2.00 |  |
| Breakfast pastries | GC | 1.00 | 1.00 | 2.00 | 0.82 |
|  | GF | 1.00 | 1.00 | 2.00 |  |

Statistical analysis was performed by comparing the points obtained by gluten-free (GF) and gluten-containing (GC) bakery products by applying only the quantitative parameters. ^1^Mann-Whitney test, *p* <0.05.
